# Successful Removal of a Posterior Cardiac Lipoma by Transection of the Ascending Aorta and Main Pulmonary Artery

**DOI:** 10.1155/2022/3813369

**Published:** 2022-08-17

**Authors:** Saeki Watanabe, Yuki Ichihara, Kozo Morita, Satoshi Saito, Hiroshi Niinami

**Affiliations:** Tokyo Women's Medical University, Department of Cardiovascular Surgery, Kawada-cho 8-1, Shinjuku-ku, Tokyo, Japan

## Abstract

Primary cardiac tumors are unusual, whereas lipomas are particularly rare. We successfully removed a very large posterior cardiac lipoma by transecting the ascending aorta and main pulmonary artery. Transecting both the ascending aorta and the main pulmonary artery facilitated surgical exposure and complete removal of the posterior cardiac lipoma.

## 1. Introduction

Primary cardiac tumors are highly unusual, and lipoma accounts for only 8.4% of these already rare tumor cases, making its occurrence extremely rare [1]. In the present case, a posterior cardiac lipoma extending into the left atrium was successfully removed by transecting the ascending aorta and main pulmonary artery, which enabled good surgical exposure and complete resection of the posterior cardiac lipoma.

## 2. Case Presentation

A 51-year-old woman presented with an abnormal electrocardiogram (ECG). The ECG showed abnormal Q waves. Further, transthoracic echocardiography revealed a substantial mass lesion on the posterior surface of the heart, and the patient was referred to our department for additional examinations.

**Figure 1:**
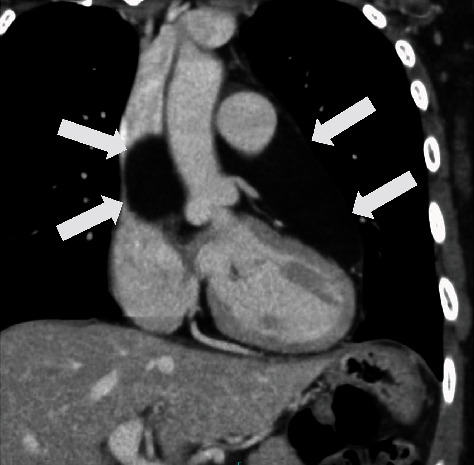
Preoperative enhanced computed tomography image. A mass on the posterior surface of the ascending aorta and main pulmonary artery is shown.

**Figure 2:**
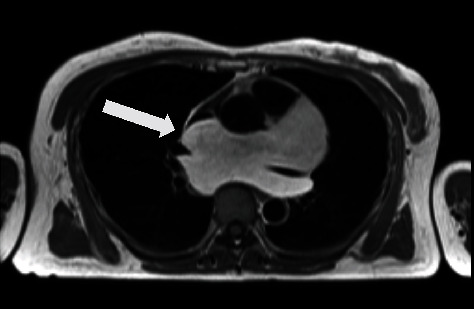
Preoperative magnetic resonance imaging findings. Arrows indicate mass.

She had no specific medical history, and blood tests showed no other abnormalities, including tumor-related parameters. ECG findings revealed a normal sinus rhythm with a heart rate of 69 bpm, no axis deviation, but abnormal Q waves at V1-V3. Transthoracic echocardiography showed a hyperechoic area sized 51 × 11 mm extending from the left atrium to the posterior wall of the left ventricle. Thoracoabdominal enhanced computed tomography (CT) revealed a homogeneous, large fatty mass extending from the posterior border of the main pulmonary artery to the dorsal left atrium, ascending aorta, and superior vena cava, which was penetrated by the bilateral pulmonary arteries (Figure 1). Magnetic resonance imaging (MRI) revealed that the mass showed high signal intensity on T1-weighted imaging (Figure 2), while it was suppressed on fat-suppressed imaging and showed low signal intensity. Coronary angiography revealed no significant stenosis of the coronary arteries and no obvious feeding vessels to the tumor. Since malignancy could not be completely ruled out and since the tumor was large enough to have the potential to compress the heart, we decided to resect the tumor completely.

**Figure 3:**
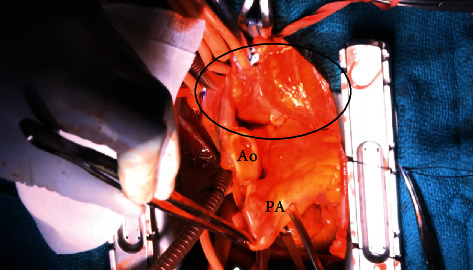
Operative image. The ascending aorta and main pulmonary artery were transected, and a mass was found on the posterior surface of the heart.

**Figure 4:**
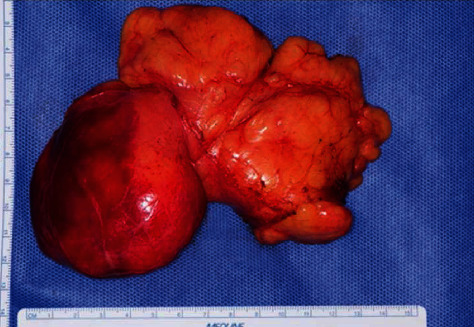
Image of the extracted mass.

The median sternotomy approach was used. The tumor was found to protrude into the left pericardial sac, extending from the dorsal surface of the superior vena cava, ascending aorta, and main pulmonary artery to the dorsal surface of the left atrium and left atrial appendage and was covered with a capsule. Total resection of the tumor by dissection of the ascending aorta and main pulmonary artery under cardiac arrest was decided. Cardiopulmonary bypass from the right femoral artery with removal from the bilateral vena cava was initiated. After the ascending aorta was cross-clamped, the ascending aorta was transected to remove the tumor from the posterior surface of the heart, and the portion on the back of the ascending aorta was dissected. Subsequently, when the main pulmonary artery was transected and the heart was removed, good exposure was obtained, and the tumor was found to be widespread on the posterior surface of the heart (Figure 3). Dissection was performed from the left atrial ceiling to the transverse pericardial sinus, and the tumor was removed as a single mass. The size was 12.5 × 7 × 4 cm (Figure 4), and an intraoperative rapid diagnosis showed no malignant component. The operation time was 294 min, with an extracorporeal circulation time of 126 min and an aortic cross-clamp time of 59 min.

**Figure 5:**
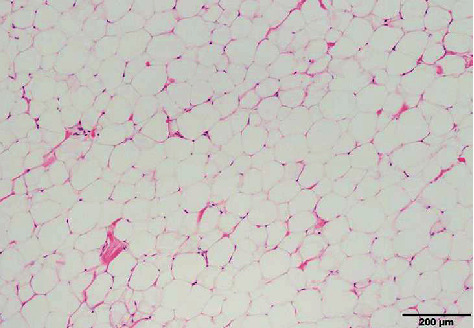
Pathological findings. The tumor was formed by hyperplasia of mature adipocytes.

Pathological findings included hyperplasia of mature adipocytes, with no hyperplasia of lipoblasts showing atypia, thus indicating a lipoma (Figure 5).

The patient was extubated on the same day as the surgery, the postoperative course was uneventful, and she was discharged from the hospital on day 12. The postoperative pathological diagnosis was lipoma.

## 3. Discussion

Primary cardiac tumors are rare, with an overall prevalence of 0.17% to 0.19% [2]. Approximately 75% of these tumors are benign, while lipoma accounts for approximately 8.4% of the cases [1]. Most lipomas are asymptomatic and often found incidentally, although depending on the site of origin, they may be symptomatic [3].

Although there were no clinical symptoms in the present case, it was decided to intervene surgically because of ECG abnormalities, a possibility of future arrhythmia, a high possibility of clinical symptoms due to its extensive presence on the posterior surface of the heart, previous reports of sudden death due to a lipoma [4], an inability to obtain a biopsy specimen from the tumor on the posterior surface of the heart, and the possibility of liposarcoma that could not be completely ruled out. Since lipomas are benign and the risk of recurrence is reported to be low [3], partial resection may be chosen if the risk of complications from tumor resection outweighs the risk of recurrence [5]. However, there are also reports of residual symptoms due to a remaining tumor and recurrence of a liposarcoma following partial resection [6], thus making complete resection desirable.

In the present case, a widespread tumor had developed on the posterior surface of the heart, and there was a concern that it might compress the organ and cause heart failure symptoms in the future. MRI findings with high signal intensity on T1- and T2-weighted images and low signal intensity on fat-suppressed images suggested the diagnosis of benign lipoma [7], while the possibility of liposarcoma could not be completely ruled out. In addition, considering that the patient was 51 years old and in an otherwise good condition, complete resection was the goal.

As mentioned earlier, the tumor was extensively widespread and was considered difficult to remove with complete resection using minimally invasive surgery. Li and Gao reported 60 (26.3%) robot-assisted tumor resections and 12 (5.3%) small right anterior lateral thoracotomy resections of primary cardiac tumors [8]; however, the minimally invasive approach was not suitable for this case because of tumor size and morphological extension. The tumor extended from the posterior margin of the superior vena cava, ascending aorta, and main pulmonary artery to the dorsal side of the left atrium, and a good surgical exposure was obtained only by transecting the ascending aorta and main pulmonary artery, thus making it possible to perform a complete resection. The ascending aorta and the main pulmonary arteries were reconstructed without any complications.

The methods used for resection in the present case are rarely employed. No residual tumor was found after the operation, and two years later, there was no recurrence.

In conclusion, complete resection of a widespread lipoma on the posterior surface of the heart was successfully performed by transecting the ascending aorta and main pulmonary artery, followed by tumor removal.

## Data Availability

All data used in this case report is included within the article.
